# A Retrospective Analysis of Mortality Due to Pneumonia and Renal Disease in a Midwestern Patient Population

**DOI:** 10.7759/cureus.73996

**Published:** 2024-11-19

**Authors:** Robert Hillard, Joselyn Schmitz, Benjamin Kossman, Lane Mittler, Vishnu Basude, Nova Beyersdorfer, Kerry Johnson, John Paulson

**Affiliations:** 1 Pathology and Anatomical Sciences, Kansas City University, Joplin, USA; 2 Medicine, Kansas City University, Joplin, USA; 3 Primary Care, Kansas City University, Joplin, USA; 4 Mathematics, Missouri Southern State University, Joplin, USA; 5 Family Medicine, Freeman Health System, Joplin, USA

**Keywords:** acute kidney injury (aki), chronic kidney disease (ckd), kidney disease, midwest, pneumonia

## Abstract

Background: The impact of pneumonia (PNA) with concomitant renal disease (RD) has not been fully investigated in a United States Midwestern patient population, despite the morbidity and mortality associated with such diseases.

Materials and methods: A retrospective cohort study was performed on International Classification of Diseases, 10^th^ Revision (ICD-10) data from a hospital system located in Southwest Missouri. Data was acquired from patients admitted between January 2019 and December 2021. Patients were separated into five groups (disease categories): acute kidney injury (AKI), chronic kidney disease (CKD), PNA, AKI with PNA, and CKD with PNA. The data were analyzed, and subset analysis was performed utilizing two-sample proportion tests (Wald test) to compare mortality rates. Informed consent was not needed due to the retrospective nature of the study.

Results: The mortality rate of patients with PNA with AKI and PNA with CKD was 36.08% (32.87% to 39.28%, 95% CI) and 24.97% (21.93% to 28.00%, 95% CI), respectively, revealing a significant increase in mortality for thosediagnosed with PNA and AKI -higher than any other disease category. For reference, PNA without (w/o) RD, CKD w/o PNA, and AKI w/o PNA had much lower mortality rates at 9.45%, 7.87% and 12.19%, respectively, with AKI w/o PNA having a 2.63% to 6.00% higher (p<0.0001) and 0.99% to 4.49% higher (p=0.0020), mortality alone than CKD w/o PNA or PNA w/o RD, respectively.

Discussion and conclusion: Mortality associated with RD and PNA was examined in a predominantly rural, relatively poor, Midwestern patient population presenting to a tertiary center with the key finding that the presence of AKI correlates with a much greater mortality rate in both patients with and without PNA. Looking forward, future studies may include a broader population base(including urban, suburban, and rural areas), allowing not only for more statistical power but also a broader assessment of the population.Such knowledge is invaluable as we continue to prioritize healthcare resources for critically ill patients suffering from RD and PNA in different settings.

## Introduction

Lower respiratory tract infections (LRTIs) are a significant cause of morbidity and mortality throughout the world. Of all the LRTIs, pneumonia (PNA) is the most significant in terms of hospitalization and mortality [1]. PNA is specifically defined as a respiratory infection that affects the “alveoli and distal airways” and can be seen in the community or hospital setting [2]. In 2019, LRTIs caused more than 2.49 million deaths [2] resulting in a 4.48% mortality globally, making it the leading cause of mortality due to infection worldwide [3]. In the United States (U.S.), per the Global Burden of Disease (GBD) study for 2019, LRTIs showed a 2.29% mortality rate. In our particular area of study, the impact was even more significant showing a 2.40% mortality rate in Missouri [3]. Although LRTI statistics allow comparison of the U.S. and Missouri to global indices (as particular causes of LRTI are not always recorded globally), if we look at PNA-specific data, 1.5% of U.S. deaths in 2019 were due to PNA [4], and, Missouri ranked 23rd and 12th for mortality out of all states for 2019 and 2020 with death rates of 13.1 and 14.3 per 100,000, respectively [5].

Not only are LRTIs and PNA a significant cause of mortality, but renal disease (RD) is as well. RD includes both acute kidney injury (AKI), also termed acute kidney disease, and chronic kidney disease (CKD), both of which drive mortality, with CKD being more significant as CKD progresses to end-stage RD (ESRD) [6], defined as kidney disease requiring permanent dialysis or transplantation [7], which results in significant mortality [8]. Such an increase in mortality is captured in epidemiological data. For example, CKD showed mortality rates of 2.57%, 4.52%, and 4.52% globally, nationally (U.S.), and locally (Missouri) per the GBD results for 2019 while acute glomerulonephritis which is a major type of AKI showed mortality rate of 0.02% across all three sets - globally, nationally (U.S.), and locally (Missouri) - during this same timeframe [3]. In addition, such evolution of CKD has a greater impact on morbidity and mortality in rural populations than in less rural areas [9]. We decided to specifically look at inpatient admissions in Missouri deriving from a large rural catchment area to analyze mortality associated with the combinatory effects of RD coupled with PNA. Given the high morbidity and mortality already known to be associated with ESRD, we focused on a more treatable area of RD coupled with PNA - namely, the mortality of AKI and CKD with PNA prior to conversion of ESRD.

In practice, underlying CKD can predispose individuals to episodes of AKI with increased severity causing decreased glomerular filtration rate [10]. Low estimated glomerular filtration rate (eGFR) (i.e., <60mL/min/1.73m2) is a general causal factor for immune suppression and the development of PNA [11]. It has been hypothesized that the mechanism of immune suppression in RD is T-cell exhaustion due to the inflammatory state produced by background uremia [12].

As CKD has been shown to preferentially affect the elderly [13], a great metric of the cost of such disease is seen in the overall Medicare costs incurred for the disease. In 2019, 87.2 billion Medicare dollars were spent on treating CKD [14]. Similarly, PNA has a great economic impact. For example, in 2021 the average 30-day cost of an episode of PNA requiring hospitalization was $14,324 [15]. As rural areas tend to be more impoverished, the economic impact of these conditions is more sorely felt. Based on available demographic data in the patients we studied, 57.6% came from rural counties as defined by the U.S. Health Resources and Services Administration [16] with the largest two counties of patients being drawn from Jasper and Newton County - in these two counties the poverty rate is 16.8% and 15.1%, respectively, compared to the national U.S. poverty rate of 11.6% based on current census data [17].

For all the prior listed reasons, a retrospective study was undertaken to identify the potential association between mortality of hospitalized patients with the combination of PNA and acute or chronic RD. Patients with PNA or acute or chronic RD without the other two conditions were also included for a reference point. Such a study adds to the literature in an often-overlooked population - namely, a patient population derived from a relatively poor, rural area in Southwest Missouri. By performing this study, we are able to create a clearer picture that these diseases have on such a demographic.

## Materials and methods

A retrospective observational cohort study was conducted on the electronic medical record database obtained from the Freeman Health System comprising the Joplin and Neosho hospitals in the Southwestern Missouri area, a non-profit tertiary referral system. Inclusion criteria included all adult patients 18 years of age or older, admitted between January 2019 and December 2021, with PNA, AKI, or CKD based on the International Classification of Diseases, 10th Revision (ICD-10) diagnostic codes (Appendix). The data was deidentified with respect to other characteristics to protect patient privacy. The study was approved through Freeman Health System's Institutional Review Board (IRB # 2022003).

The patient data were then categorized into five unique groups (Table 1). Mortality rates were calculated for each group as defined as the percentage of deaths that occurred over the study time period. The data were statistically analyzed using the Wald tests and two sample proportion tests with confidence intervals for the proportional difference. Exclusion criteria included multiple admissions for the same disease, patients with ESRD, or patients with comorbidities not within the scope of this study.

**Table 1:** Pneumonia and renal disease groups. This table describes five groups of patients created from the combinations of International Classification of Diseases, 10th Revision (ICD-10) codes assessed.

Pneumonia and Renal Disease Groups	
P1	Pneumonia (PNA) with chronic kidney disease (CKD)
P2	Pneumonia with acute kidney injury (AKI)
P3	Pneumonia without renal disease (RD)
P4	Chronic kidney disease without pneumonia
P5	Acute kidney injury without pneumonia

**Figure 1:**
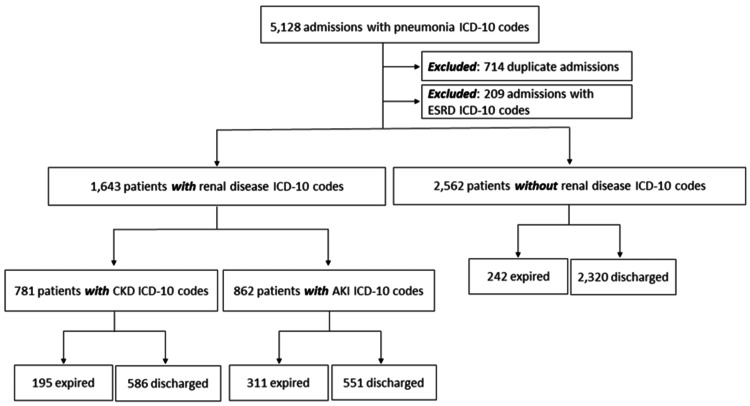
Flowchart detailing patient stratification with inclusion and exclusion criteria of adult patients admitted from January 2019 to December 2021. ICD-10 = International Classification of Diseases, 10th Revision; ESRD = end-stage renal disease; CKD = chronic kidney disease; AKI = acute kidney injury.

There were 5,128 patients identified with the PNA ICD-10 code. Of those, 714 patients were excluded from the data due to a duplicate admission, and 209 patients were excluded due to ESRD. The remaining 4,205 patients were then split based on presence and type of RD and mortality was assessed in these subgroups (Figure 1). Note that those categorized as having CKD also included patients that may have had AKI superimposed on CKD but those categorized as having acute renal disease, i.e., AKI, had AKI only.

**Figure 2:**
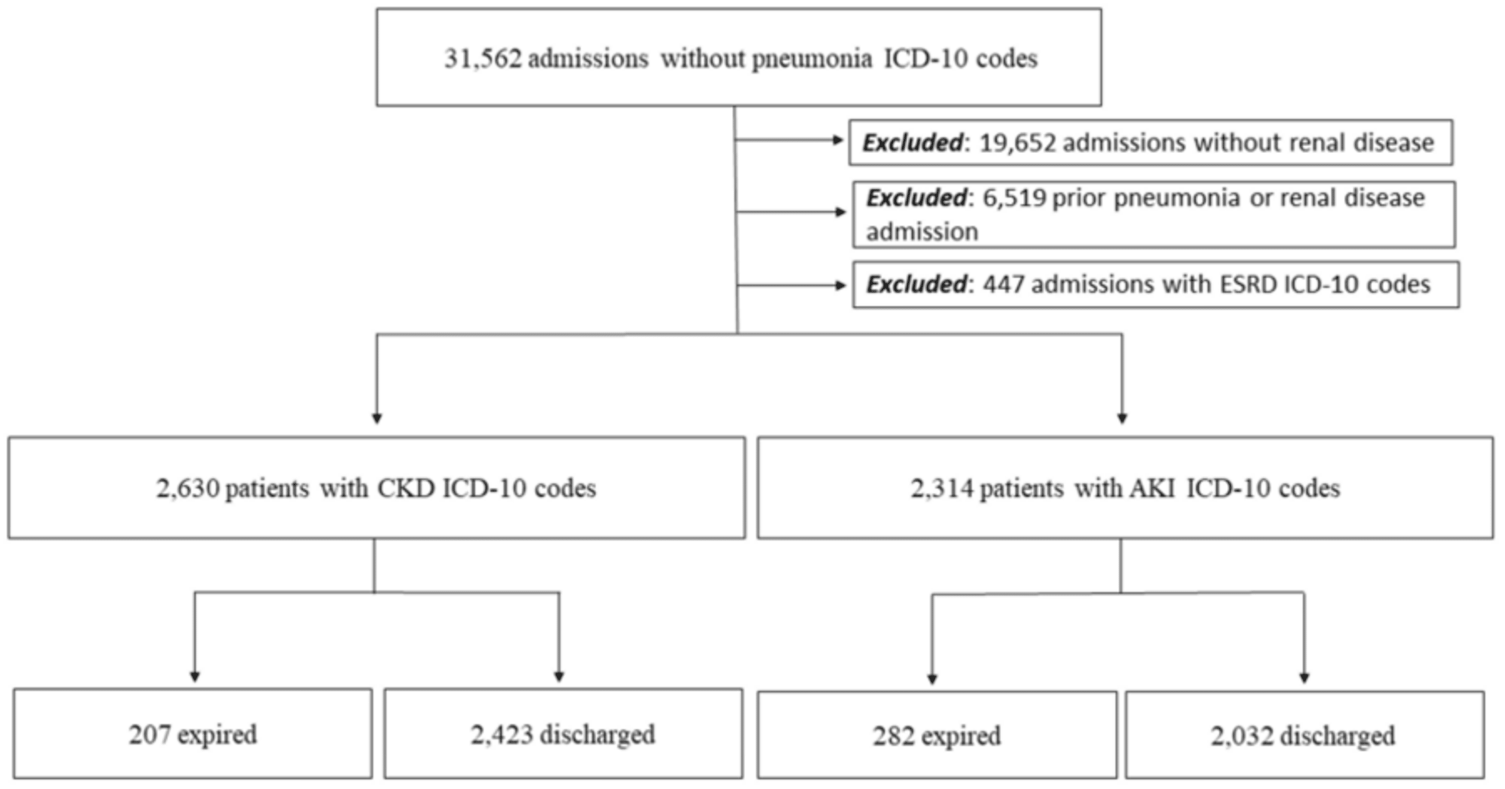
Flowchart detailing patient stratification with inclusion and exclusion criteria of adult patients admitted from January 2019 to December 2021. ICD-10 = International Classification of Diseases, 10th Revision; ESRD = end-stage renal disease; CKD = chronic kidney disease; AKI = acute kidney injury.

For the analysis of outcomes in patients without PNA, 31,562 admissions lacking the PNA ICD-10 codes were identified. From this group, patients without ICD-10 codes for RD (19,652) were excluded. In addition, patients with prior RD hospitalizations or PNA hospitalizations (6,519) or current ESRD (447) were also excluded from the analysis (Figure 2).

## Results

**Figure 3:**
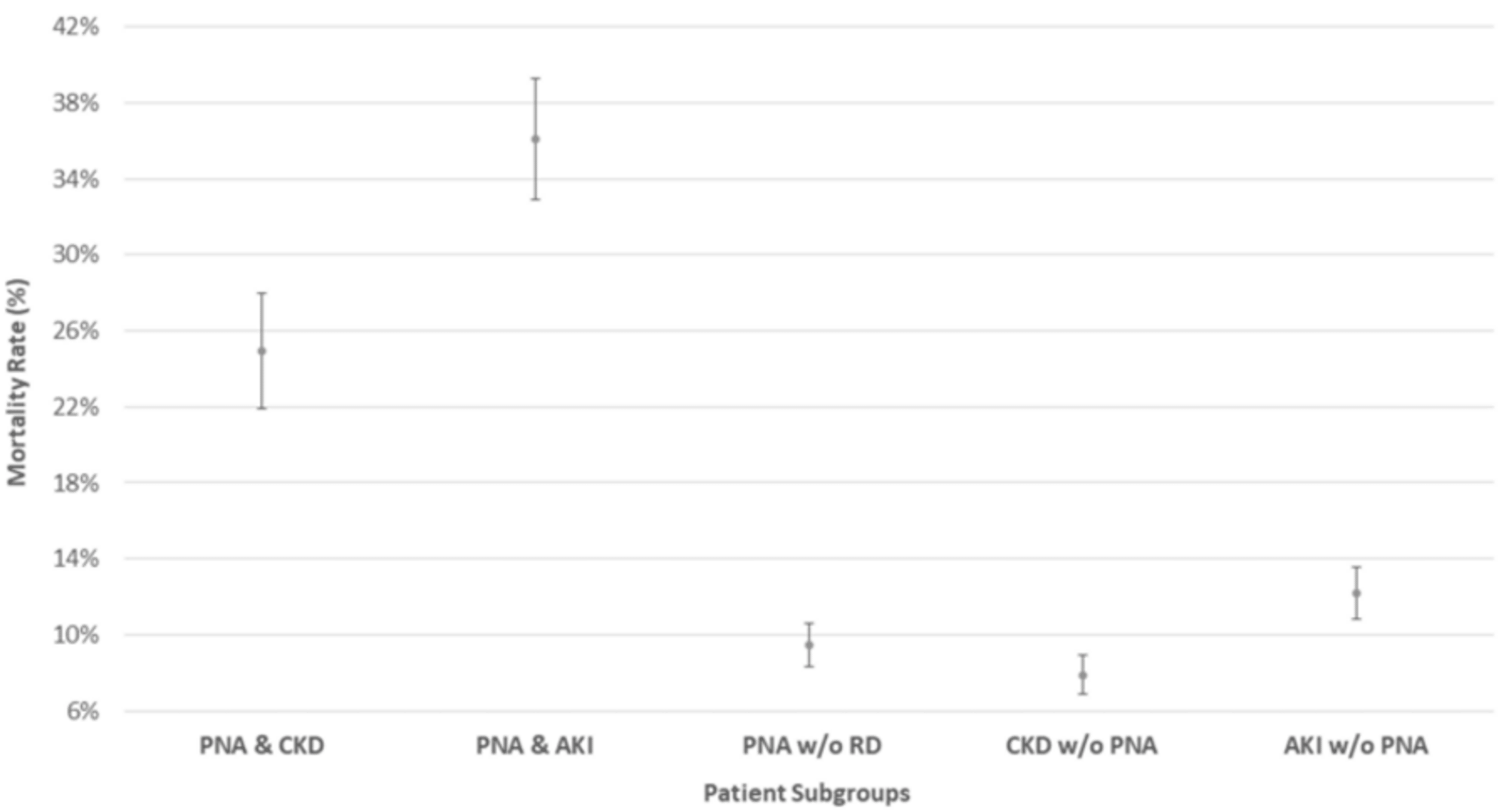
Mortality rates for subgroup population with 95% confidence intervals. PNA = pneumonia, CKD = chronic kidney disease, AKI = acute kidney injury, RD = renal disease, w/o = without.

The majority of patients who met the inclusion criteria were male (53.6% male; 46.4% female) and predominantly 65 years old or greater (63.4% 65 years old or greater; 36.6% under 65 years old). In general, we found that PNA coupled with either type of RD had a much higher mortality rate than any condition alone. Specifically, the mortality rate of patients with PNA with CKD and PNA with AKI was 24.97% (21.93% to 28.00%, 95% CI) and 36.08% (32.87% to 39.28%, 95% CI), respectively, revealing a significant increase in mortality for those diagnosed with PNA and AKI - higher than any other disease category. For reference, PNA without (w/o) RD, CKD w/o PNA, and AKI w/o PNA had much lower mortality rates at 9.45%, 7.87%, and 12.19%, respectively (Figure 3). The relative ranking of mortality rate with respect to disease category was unchanged regardless of advanced age (65 years old or greater) or sex.

If we look in particular at AKI, we found it was associated with an increase in mortality: it not only had a mortality rate that was higher than either CKD or PNA alone, 2.63% to 6.00% higher (p<0.001) and 0.99% to 4.49% higher (p=0.0020), respectively, but more importantly when coupled with PNA had a mortality rate which was 6.70% to 15.53% (p<0.0001) higher than the second highest mortality cause (i.e., CKD + PNA). In regard to CKD with PNA, it showed a 9.47% to 16.10% higher mortality than the third highest cause of mortality which was AKI without PNA (p<0.0001) (Table 2).

**Table 2:** Comparison (two-sample proportion test and Wald test) of mortality rates of the study subpopulations. * = The rows within both of these columns give the mortality sample of the groups. The numerator represents the total mortality in each particular sample group with the denominator representing the total number of the sample group. The number below the fraction represents the sample proportion (i.e., result of the division). ** = This column gives the absolute difference between the sample proportions of the two particular groups. P1 = pneumonia with chronic kidney disease; P2 = pneumonia with acute kidney injury; P3 = pneumonia without renal disease; P4 = chronic kidney disease without pneumonia; P5 = acute kidney injury without pneumonia.

Comparison sample 1 vs sample 2	Mortality sample 1*	Mortality sample 2*	Absolute difference**	Lower 95% CI for P1-P2	Upper 95% CI for P1-P2	P-value
P1 vs P2	195/781	311/862	0.1111	0.0670	0.1553	<0.0001
	0.2497	0.3608				
P1 vs P3	195/781	242/2,562	0.1552	0.1228	0.1876	<0.0001
	0.2497	0.0945				
P1 vs P4	195/781	207/2,630	0.1710	0.1389	0.2030	<0.0001
	0.2497	0.0787				
P1 vs P5	195/781	282/2,314	0.1278	0.0947	0.1610	<0.0001
	0.2497	0.1219				
P2 vs P3	311/862	242/2,562	0.2663	0.2323	0.3003	<0.0001
	0.3608	0.0945				
P2 vs P4	311/862	207/2630	0.2821	0.2484	0.3158	<0.0001
	0.3608	0.0787				
P2 vs P5	311/862	282/2,314	0.2389	0.2042	0.2736	<0.0001
	0.3608	0.1219				
P3 vs P4	242/2,562	207/2630	0.0158	0.0004	0.0311	0.0435
	0.0945	0.0787				
P3 vs P5	242/2,562	282/2,314	0.0274	0.0099	0.0449	0.0020
	0.0945	0.1219				
P4 vs P5	207/2,630	282/2,314	0.0432	0.0263	0.0600	<0.0001
	0.0787	0.1219				

## Discussion

The finding that the mortality rate of patients with AKI coupled with PNA is highest compared to all other subgroups when ESRD is excluded is not surprising as previously published results show an increased mortality rate of individuals with PNA and coexisting acute renal disease [18,19]. One potential pathophysiologic mechanism that may exacerbate acute renal disease in the background of PNA is the presence of an increased proinflammatory cytokine state [19] which can lead to a buildup of reactive oxygen species and cause direct kidney damage [20]. In addition, hypoxemia has been shown to affect the loops of Henle and proximal tubules in animal models [21,22], and, more specifically, hypoxia is thought to contribute to AKI (through acute tubular injury) in COVID-19 patients which may have a concomitant respiratory illness, including PNA [23].

The higher levels of mortality seen in AKI patients without a history of CKD when seen in isolation or when paired with PNA is not unexpected as many studies in the past have documented exceedingly high rates of in-hospital mortality in patients with AKI - often exceeding 50% in critically ill patients [24]. Furthermore, patients who develop AKI within the hospital setting without superimposed CKD have been found to have worse outcomes than patients with previous CKD, which may be attributable to a lower level of insult needed to cause AKI in CKD patients when compared to those with only AKI [10]. Alternatively, some have hypothesized that CKD has upregulated molecular pathways that drive a protective response in a chronically damaged kidney such as through the modulation of metabolic, angiogenic, and cell proliferation responses by hypoxia-inducible factor [25]. Regardless of the underlying mechanism, based on our results, the subset of critically ill patients with PNA and AKI fared the worst in our study population.

Finally, it should be noted that the exclusion of patients with ESRD, allowed us to focus on the mortality of disease states that still had some degree of renal function, not necessitating permanent dialysis or transplantation (i.e. not ESRD). Such a focus allowed us to show differences in the effects of PNA on mortality in potentially correctable causes of RD. In this particular subset, AKI, not CKD, appeared to be the driver of mortality.

The results contribute to the existing literature on RD not only by focusing on individuals with PNA but also by reporting on a patient population that encompasses a rural, more impoverished demographic. Aside from the fact that poorer individuals are more hesitant to seek out medical care and thus present with more severe disease conditions, another factor that impacts kidney function in rural settings is that agricultural pesticides often contain nephrotoxic chemicals. For example, glycine phosphonate, which is utilized in certain pesticides, can combine with heavy metals (cadmium, lead, etc.) producing a stable nephrotoxic compound. Alternately, well water often contains metal cations (calcium, magnesium, iron) which have been thought to combine with agricultural compounds causing nephrotoxicity [26]. Lastly, it should be noted that in addition to CKD, AKI has also been seen in higher levels of agricultural workers. For example, in one study involving California field workers, it was shown that there was a higher incidence of AKI after only a single day of labor [27]. Although such specific risk factors for our patient population were not included in our current study, such considerations may form the basis for further research.

Limitations to this study include the fact that it was a retrospective study therefore the sample was not chosen at random. As this is the case, it is unable to be determined if the sample analyzed is representative of the entire population. Furthermore, due to the specific sample chosen, our study population did not afford the statistical power to evaluate additional comorbid conditions.

## Conclusions

Mortality due to RD and PNA was examined in a unique resource-challenged Midwestern rural population with the key finding that the presence of AKI correlates with a much greater mortality rate in both patients with and without PNA. Looking forward, future studies may include a broader population base (including urban, suburban, and rural areas), allowing not only for more statistical power but also a broader assessment of the population. Such knowledge is invaluable as we continue to prioritize healthcare resources for critically ill patients suffering from RD and PNA in different settings.

## Appendices

**Table 3:** ICD-10 codes for patients with pneumonia, acute kidney injury, and chronic kidney disease.

Pneumonia ICD-10 Codes	Diagnosis
J1000	Influenza due to other identified influenza virus with unspecified type of pneumonia
J1001	Influenza due to other identified influenza virus with the same other identified influenza virus pneumonia
J1008	Influenza due to other identified influenza virus with other specified pneumonia
J1100	Influenza due to unidentified influenza virus with unspecified type of pneumonia
J1108	Influenza due to unidentified influenza virus with specified pneumonia
J120	Adenoviral pneumonia
J121	Respiratory syncytial virus pneumonia
J122	Parainfluenza virus pneumonia
J123	Human metapneumovirus pneumonia
J1281	Pneumonia due to SARS-associated coronavirus
J1282	Pneumonia due to coronavirus disease 2019
J1289	Other viral pneumonia
J129	Viral pneumonia, unspecified
J13	Pneumonia due to Streptococcus pneumoniae
J14	Pneumonia due to Hemophilus influenzae
J150	Pneumonia due to Klebsiella pneumoniae
J151	Pneumonia due to Pseudomonas
J1520	Pneumonia due to staphylococcus, unspecified
J15211	Pneumonia due to Methicillin susceptible Staphylococcus aureus
J15212	Pneumonia due to Methicillin resistant Staphylococcus aureus
J1529	Pneumonia due to other staphylococcus
J153	Pneumonia due to streptococcus, group B
J154	Pneumonia due to other streptococci
J155	Pneumonia due to Escherichia coli
J156	Pneumonia due to other Gram-negative bacteria
J157	Pneumonia due to Mycoplasma pneumoniae
J158	Pneumonia due to other specified bacteria
J159	Unspecified bacterial pneumonia
J168	Pneumonia due to other specified infectious organisms
J17	Pneumonia in diseases classified elsewhere
J180	Bronchopneumonia, unspecified organism
J181	Lobar pneumonia, unspecified organism
J188	Other pneumonia, unspecified organism
J189	Pneumonia, unspecified organism
J84116	Cryptogenic organizing pneumonia
J851	Abscess of lung with pneumonia
J95851	Ventilator associated pneumonia

Acute Kidney Injury ICD-10 Codes	Diagnosis
N170	Acute kidney failure with tubular necrosis
N178	Other acute kidney failure
N179	Acute kidney failure, unspecified

Chronic Kidney Disease ICD-10 Codes	Diagnosis
N181	Chronic kidney disease, stage 1
N182	Chronic kidney disease, stage 2 (mild)
N183	Chronic kidney disease, stage 3 (moderate)
N1830	Chronic kidney disease, stage 3 unspecified
N1831	Chronic kidney disease, stage 3a
N1832	Chronic kidney disease, stage 3b
N184	Chronic kidney disease, stage 4 (severe)
N185	Chronic kidney disease, stage 5
N189	Chronic kidney disease, unspecified
